# Stability of population genetic structure in large yellow croaker (*Larimichthys crocea*): Insights from temporal, geographical factors, and artificial restocking processes

**DOI:** 10.1002/ece3.70207

**Published:** 2024-08-27

**Authors:** Ji‐Xiang Yao, Hung‐Du Lin, Li‐Sheng Wu, Li‐Na Wu, Ji‐Gui Yuan, Shao‐Xiong Ding

**Affiliations:** ^1^ State Key Laboratory of Marine Environmental Science, College of Ocean and Earth Sciences Xiamen University Xiamen China; ^2^ The Affiliated School of National Tainan First Senior High School Tainan Taiwan

**Keywords:** artificial restocking, COI, cyt *b*, *Larimichthys crocea*, population genetic structure

## Abstract

Despite concerns about overfishing and the potential impact of release programs on wild populations, our study of 3116 individuals from 13 wild populations and 2787 individuals from two cultured populations in Zhejiang and Fujian provinces spanning 2008 to 2023 reveals a relatively stable genetic diversity in *Larimichthys crocea*. Surprisingly, the genetic diversity of wild large yellow croaker populations has remained consistent over the years, suggesting minimal influence from population declines due to overfishing. With the exception of populations in Sansha Bay and Luoyuan Bay, no significant genetic differences were observed among wild populations, indicating a single panmictic genetic population across the East and South China seas. Notably, significant genetic differentiation exists between cultured and wild populations, suggesting a possible limited genetic adaptation of cultured‐released individuals to the wild environment. The genetic differences observed between the Sansha Bay, with its adjacent Luoyuan Bay populations, and other wild populations underscore the dual effects of habitat environment and farming activities on the genetic structure of large yellow croaker. Our findings suggest that, despite declines in population numbers due to overfishing and expands extensive cultured releases, the genetic diversity of *L. crocea* populations remains largely unaffected. Moreover, the *L. crocea* population along the Chinese coast appears to form a single panmictic population with considerable genetic diversity.

## Graphical abstract

The genetic diversity of wild large yellow croaker populations has remained consistent over the years, suggesting minimal influence from population declines due to overfishing. A limited genetic adaptation of cultured‐released individuals to the wild environment. Moreover, the *L. crocea* population along the Chinese coast appears to form a single panmictic population with considerable genetic diversity.

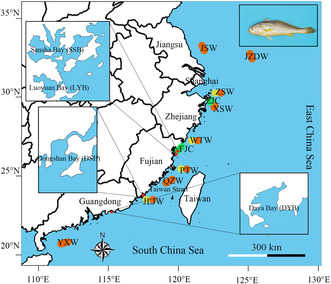

## 1 INTRODUCTION

With the global population rapidly increasing, seafood has become a critical food source. However, overfishing has precipitated a decline in global fishery resources (Madduppa et al., 2018; Srinivasan et al., 2010; Sumaila et al., 2016; Zhang et al., 2023). Developed fish species are particularly vulnerable to population declines and environmental changes due to global shifts, potentially resulting in reduced genetic diversity and diminished adaptability to current and future environmental challenges (Petit‐Marty et al., 2022). To address this issue, countries and regional fisheries management organizations (RFMOs) have implemented management and governance measures, including extensive stock enhancement efforts (Herrera et al., 2023; Paulsen & Støttrup, 2004; Purcell et al., 2004; Sumaila et al., 2016; Wang et al., 2021; Zohar et al., 2008). For instance, China has expanded fishing bans in coastal areas and released juvenile individuals of different species to bolster wild populations (Wang et al., 2021).

Large yellow croakers (*Larimichthys crocea*) have garnered considerable research attention due to their broad market demand and successful artificial reproduction (Wang et al., 2012; Wu et al., 2014). But overfishing has led to a significant decline in their populations (Liu & De Mitcheson, 2008; Xu et al., 2022; Yuan et al., 2021), with the wild population experiencing collapse (Liu & De Mitcheson, 2008). In response, the Chinese government has initiated a comprehensive program aimed at replenishing large yellow croaker populations. Since overcoming technical challenges in artificial propagation in 1985, large‐scale fry production has commenced, reaching one billion fry by 2000 (Zhang et al., 2022). By 2014, annual production had reached 127,917 tons, making large yellow croaker the most cultured marine fish species in China, with 92.400% of production concentrated in Fujian and Zhejiang provinces (The People's Republic of China Ministry of Agriculture, Fisheries Bureau, 2015; Yang et al., 2022).

As an economically important species in both fisheries and aquaculture, extensive research has been conducted on wild and cultured populations of large yellow croaker. Understanding their phylogeographic patterns and genetic diversity is crucial for effective conservation. The configuration of the marginal seas in the northwestern Pacific underwent dramatic changes during the Pleistocene glacial–interglacial cycles. The formation of the Taiwan Strait, due to sea‐level drops of approximately 120–140 m during glacial periods, likely served as a phylogeographic barrier, hindering the dispersal of marine fish between the strait's opposite sides. Previous studies reveal the presence of two distinct lineages in some marine fish species, such as the Chinese black sleeper (*Bostrychus sinensis*) (Qiu et al., 2016), cutlassfish (*Trichiurus nanhaiensis*) (Gu et al., 2022) and Yellow Grouper (*Epinephelus awoara*) (Yang et al., 2022), as a result of these vicariance events. While early studies classified large yellow croaker into distinct populations based on morphology, subsequent molecular genetic research suggests limited geographical isolation among wild populations (Han et al., 2015; Wang et al., 2013; Yuan et al., 2021; Zhang et al., 2023). However, some studies still suggest genetic differentiation, particularly based on SNPs (Chen et al., 2023; Jiang et al., 2015; Kon et al., 2021). Our recent results indicate the presence of a single panmictic population in the large yellow croaker and suggest that the vicariant effect of sea level decreases in the Taiwan Strait may not promote its divergence (Liu et al., 2020; Yuan et al., 2021, 2024). Notably, genetic diversity within cultured populations is lower than in wild populations, highlighting significant distinctions between them (Guo et al., 2005; Liu et al., 2020; Yuan et al., 2021). To address gaps in understanding wild geographic populations' genetic diversity and structure, this study collected samples from wild populations across their distribution range, alongside cultured populations from Zhejiang and Fujian. Analyzing genetic temporal sequence data from historical samples and integrating genetic variables are crucial for conservation efforts. Despite overfishing threats, recent studies indicate sustained high genetic diversity in large yellow croaker populations (Xu et al., 2022; Yuan et al., 2021). However, the impact of cultured practices on genetic composition warrants investigation.

Mitochondrial genetic markers offer insights into genetic diversity and conservation status, yet prior efforts haven't comprehensively assessed these aspects for large yellow croaker populations. Thus, this study aims to bridge this gap by examining the genetic diversity of multiple geographic populations, changes over time, genetic differences across locations, and the impact of releases on wild populations and genetic structure among cultured populations. These findings will inform fishery management practices and aid in developing effective conservation strategies.

## 2 MATERIALS AND METHODS

### 2.1 Sample collection

**FIGURE 1:**
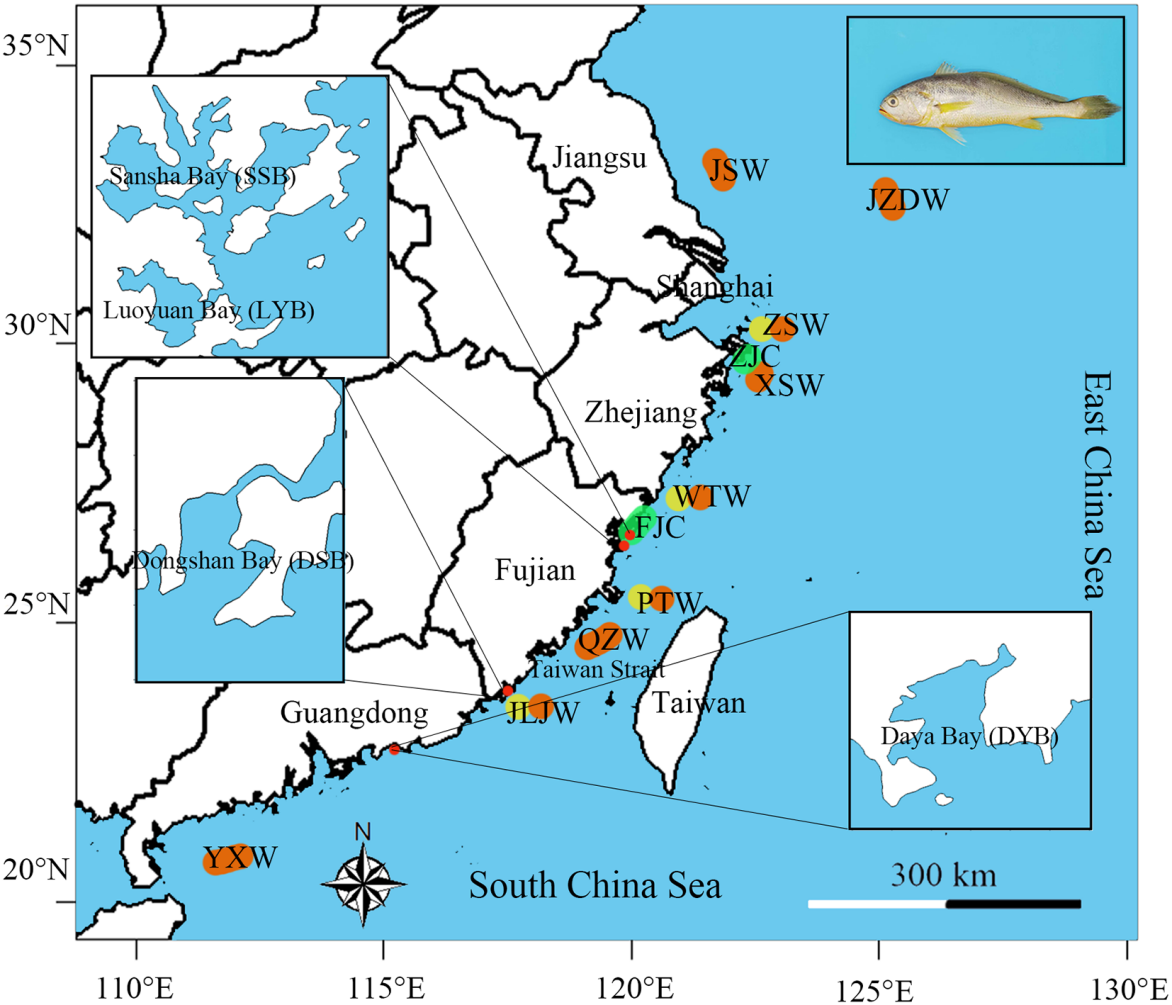
Map showing sampling locations of the large yellow croaker. JSW: Wild population collected from sea areas near Jiangsu province; JZDW: Wild population collected near Jeju Island, ZSW: Wild population collected near Zhoushan Island, XSW: Wild population collected near Yushan Islands, WTW: Wild populations from the sea area bordering Zhejiang and Fujian province, PTW: Wild population collected near Haitan Island, QZW: Wild population collected from sea areas near Quanzhou city, JLJW: Wild population collected near Kinmen Island, YXW: Wild population collected near the Leizhou Peninsula. SSB, LYB, DSB, and DYB: Wild populations collected from Sansha Bay, Luoyuan Bay, Dongshan Bay, and Daya Bay, respectively. ZJC and FJC: Cultured populations collected from most of Zhejiang and Fujian province.

Thirteen wild populations included nine populations (JSW, JZDW, ZSW, XSW, WTW, PTW, JLJW, QZW, and YXW) collected samples from 2008, 2016 to 2023 in the open sea using trawler and/or drift nets and four populations (SSB, LYB, DSB, and DYB) collected in the bay (Figure 1 and Table 1). In this study, we sampled a total of 3116 individuals from 13 wild populations. Among these, 571 samples were from a study conducted by Yuan et al. (2021), while 2545 samples were collected for this study. We also collected two cultured populations (ZJC and FJC) from Zhejiang and Fujian provinces in 2018 and 2019. From these cultured populations, we sampled a total of 2787 individuals, with 2372 from FJC and 367 from ZJC as reported by Yuan et al. (2021). Additionally, 48 new samples from ZJC were included in this study. A small piece of muscle tissue or a fin clip from each sample was preserved in 95.000% ethanol at −20°C to aid in the subsequent DNA extraction process.

**TABLE 1:** Sampling locations and summary statistics of the large yellow croaker.

Location	Code name	Sample size	Year of sampling	Sampling times	Private haplotype, Ph	Number of haplotypes, H	Haplotype diversity, Hd	Nucleotide diversity, θπ/%
Zhejiang Cultured	ZJC	415	2017–2019, 2021	14	3	19	0.509	0.129
Fujian Cultured	FJC	2372	2017–2019	106	8	30	0.732	0.257
Jiangsu	JSW	142	2006, 2008, 2019, 2021, 2022	7	37	80	0.987	0.414
Jeju Island	JZDW	89	2022	6	30	70	0.993	0.448
Zhoushan Island	ZSW	193	2018, 2019, 2022	1	35	91	0.982	0.460
Yushan Islands	XSW	99	2019–2022	5	27	69	0.988	0.455
Wenzhou and Fuding	WTW	306	2020–2023	10	60	140	0.979	0.410
Sansha Bay	SSB	756	2017, 2019–2023	20	10	38	0.784	0.267
Luoyuan Bay	LYB	58	2022–2023	2	4	20	0.874	0.291
Haitan Island	PTW	243	2020–2023	12	45	116	0.986	0.436
Quanzhou	QZW	67	2021–2023	4	10	50	0.988	0.421
Kinmen Island	JLJW	667	2017, 2018, 2020–2023	26	192	298	0.991	0.436
Dongshan Bay	DSB	84	2020–2022	6	13	59	0.985	0.393
Daya Bay	DYB	291	2021–2023	7	62	151	0.990	0.438
Leizhou Peninsula	YXW	121	2016, 2018, 2022	5	39	82	0.989	0.459
Cultured total		2787		120	25	30	0.707	0.241
Wild total		3116		111	688	725	0.887	0.338

### 2.2 Primer design and PCR amplification

In this study, the mitochondrial genes COI and cyt *b* fragment sequences were amplified by designing a specific primer (Yuan et al., 2021). The PCR amplification was carried out using a 25 μL system with Tengen 2× Taq PCR Mastermix (KT201‐01), following the specified protocol. The PCR reaction mixture consisted of 12.500 μL of 2× Taq PCR Mastermix, 8 μL of sterile water, and 1 μL each of the forward and reverse primers. For the COI gene amplification, the PCR reaction conditions were as follows: an initial denaturation at 94°C for 5 min, followed by 30 cycles at 94°C for 30 s, 52°C for 30 s, and 72°C for 1 min, and followed at 72° for 10 min. Similarly, for the cyt *b* gene amplification, the PCR reaction conditions were as follows: an initial denaturation at 94°C for 5 min, followed by 35 cycles at 94°C for 30 s, 52.500°C for 30 s, and 72°C for 2 min, and followed at 72° for 10 min. The PCR products were stored in a refrigerator at 4°C until further processing. Finally, the products were sent to Sangon Biotech (Shanghai) for sequencing.

### 2.3 Population diversity and structure

After sequencing, the polymorphic sites in the fragment sequences were manually inspected and corrected by referring to the corresponding chromatograms using Sequencher software (Tippmann, 2004). The sequences were aligned using Mega 11 (Tamura et al., 2021) and then optimized manually. A grand total of 5903 unified fragments (COI + cyt *b*) were employed to compute genetic diversity indices—including the number of haplotypes (H), private haplotype (Ph), haplotype diversity (Hd) (Nei & Tajima, 1983), and nucleotide diversity (θπ) using DNAsp v6 software (Rozas et al., 2017) to assess genetic diversity levels within each population. Genetic distances among the populations were calculated using the Kimura 2‐Parameter model in MEGA 11 software program, and a neighbor‐joining (NJ) tree was constructed for all populations. PCoA (principal co‐ordinates analysis) was used to characterize the distance relationship between cultured populations and plotted it using R.

We define the divergent three aquatic regions based on topography, 30 m water depth and fishing practices (included trawling and drift gillnet) and marked in Figure 1, where red circle means inner bay, yellow circle means inshore, and orange circle means offshore. The inner populations are samples collected from concave portions of the coastline or protruding portions of the ocean. The inshore population comprises samples collected in areas with water depths shallower than 30 meters, primarily captured using drift nets. Conversely, the offshore population includes samples inhabiting sea areas with water depths exceeding 30 meters and typically caught using trawling methods. To compare genetic diversity among regions, we conducted an analysis using one‐way analysis of variance (ANOVA) using IBM SPSS Statistics 24. The aim was to compare the mean genetic diversity among the target populations to determine if there were statistical differences between the target population and other populations.

To estimate genetic differentiation between populations, global and pairwise *F*_ST_ values were calculated with the Weir and Clark Cockerham method (Weir & Cockerham, 2023) using Arlequin 3.5 (Excoffier & Lischer, 2010). The significance of these values was estimated with 10,000 permutations. *F*_ST_ values aggregated with genetic distances, drawn and exported using OriginPro (Version 2023b, OriginLab Corporation, Northampton, MA, USA). Finally, a hierarchical analysis of molecular variance (AMOVA) was performed with 1000 random permutations in Arlequin 3.5 (Excoffier & Lischer, 2010) to partition the genetic structure and source of variation of the populations, and the proportion of total variation within and among populations was calculated. For AMOVA (Dupanloup et al., 2002; Excoffier et al., 1992), the wild populations were divided into three scenarios according to inner bay populations and others, restocking intensity and geographical populations: (1) Scenario I: two independent groups including the inner bay populations (SSB, LYB, DSB, DYB) and others (JSW, JZDW, ZSW, XSW, WTW, PTW, QZW, JLJW, YXW), representing the inner bay populations and other populations, respectively; (2) Scenario II: two independent groups including the populations closed to cultured populations (SSB, LYB) and other populations (DSB, DYB, JSW, JZDW, ZSW, XSW, WTW, PTW, QZW, JLJW, YXW), based on a NJ tree; (3) Scenario III: three groups including (i) Daiquyang stock (ranging from the southern Yellow Sea to central East China Sea, (JSW, JZDW, ZSW, XSW)); (ii) Min‐Yuedong stock (ranging from the southern East China Sea to eastern Pearl River, (WTW, PTW, QZW, JLJW)); (iii) Naozhou stock (ranging from the western Pearl River to Leizhou Peninsula, (YXW)), which were primarily divided by geographic groups based on Tian et al. (1962). (4) Scenario IV: three groups including (i) inner bay populations (SSB, LYB, DSB, DYB); (ii) inshore populations (ZSW, WTW, PTW, JLJW); (iii) offshore populations (JSW, JZDW, ZSW, WTW, PTW, JLJW, YXW), based on aquatic regions.

## 3 RESULTS

### 3.1 Genetic diversity and population genetic structure of large yellow croaker wild populations

**FIGURE 2:**
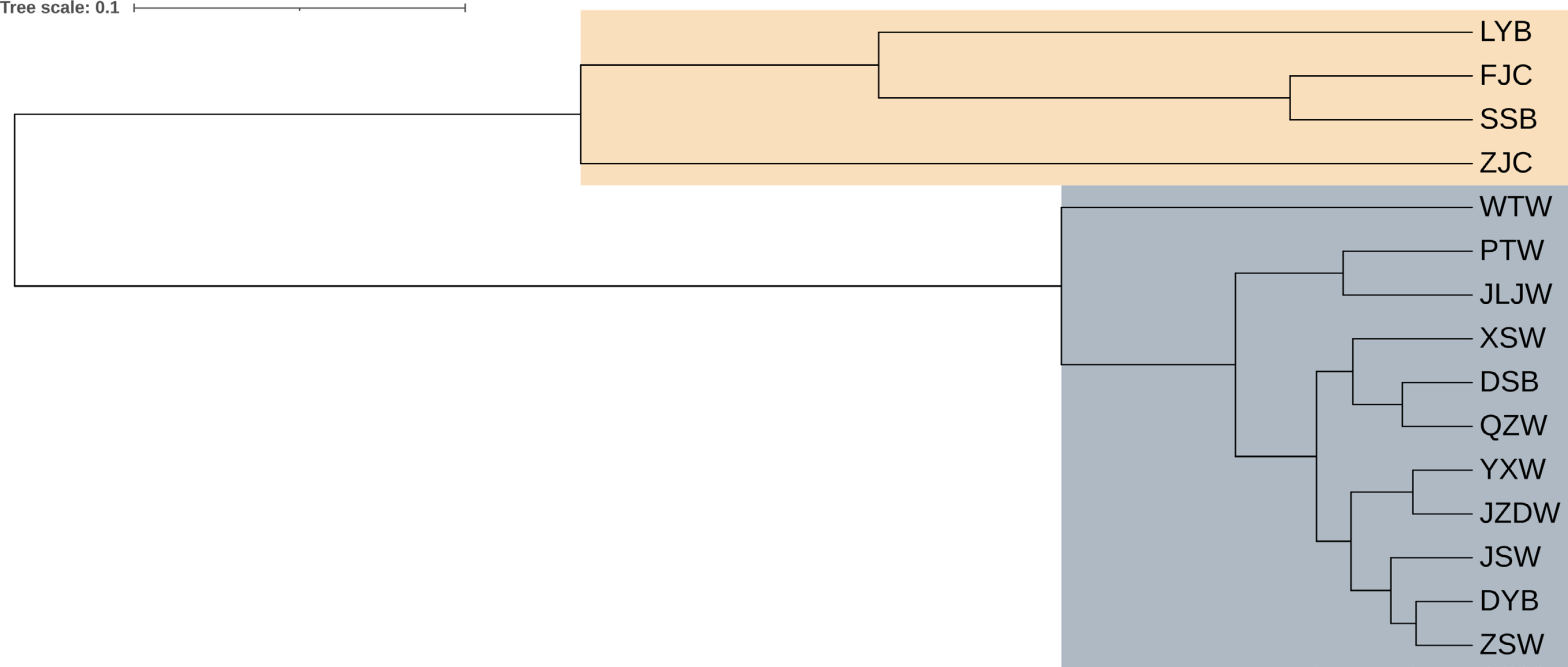
Neighbor‐joining tree of mtDNA (COI + cyt *b*) sequences in the large yellow croaker. Yellow color indicates the cultured populations, SSB and LYB are clustered together, gray color indicates the 11 wild populations are clustered together.

A total of 1657 haplotypes (1657 bp; cyt *b*, 1055 bp and COI, 602 bp) were obtained for the 3116 specimens from the 13 wild populations and two cultured populations analyzed (Table 1 and Figure 1). Overall, the average haplotype diversity (Hd) among wild populations was 0.959, ranging from 0.784 (SSB) to 0.993 (JZDW) within specific populations. In the cultured population, the average diversity (Hd) stood at 0.707, varying from 0.509 (ZJC) to 0.732 (FJC) within populations. The average nucleotide diversity (θπ) among wild populations was 0.389%, ranging from 0.267% (SSB) to 0.460% (ZSW), while in cultured populations, it stood at 0.241%, varying between 0.129% (ZJC) and 0.257% (FJC). Notably, the SSB and LYB populations exhibit lower haplotype and nucleotide diversities among wild populations, while the remaining populations maintain similar levels of diversity. The genetic diversity parameters, including the number of haplotypes, haplotype diversity, and nucleotide diversity, were calculated for each population and are presented in Table 1. Neighbor‐joining (NJ) tree analysis revealed two primary lineages. Eleven wild populations clustered within one lineage, while the SSB and LYB wild populations grouped with the cultured populations, forming the other lineage (Figure 2). This clustering suggests some level of genetic similarity between the SSB, LYB, and the cultured populations, while the other wild populations maintain distinct genetic lineages. The pairwise *F*_ST_ values indicated low genetic differentiation among wild populations, ranging from 0.000 to 0.114. The overall standardized *F*_ST_ value across all wild populations was 0.021. Supporting the phylogenetic analysis, the genetic distances, and *F*_ST_ values also confirm genetic differentiation among the SSB, LYB, and other wild populations (ranging from 0.016% to 0.317%) (Figure S1). Hierarchical analyses of molecular variance (AMOVA) from the two groups based on phylogenetic tree (Scenario II, SSB + LYB and other wild populations) demonstrated that significant spatial genetic structuring among groups was 7.230% (*F*_CT_ = 0.072, *p* = .013) but was only 0.120% (*F*_SC_ = 0.001, *p* = .136) among populations within groups and 92.650% (*F*_ST_ = 0.074, *p* < .000) within populations (Table 2).

**TABLE 2:** Analysis of molecular variance (AMOVA) of the large yellow croaker based on mtDNA.

Scenario	Source of variation	Percentage of variation	Static	*p*
Scenario I:Two groups (SSB, LYB, DSB, DYB) (JSW, JZDW, ZSW, XSW, WTW, PTW, QZW, JLJW, YXW)	Among groups	3.340	FCT = 0.033	.082
	Among populations within groups	2.200	FSC = 0.022	.000
	Within populations	94.460	FST = 0.055	.000
Scenario II:Two groups (SSB, LYB) (DSB, DYB, JSW, JZDW, ZSW, XSW, WTW, PTW, QZW, JLJW, YXW)	Among groups	7.230	FCT = 0.072	.013
	Among populations within groups	0.120	FSC = 0.001	.136
	Within populations	92.650	FST = 0.074	.000
Scenario III:Three groups (JSW, JZDW, ZSW, XSW, WTW) (PTW, QZW, JLJW) (YXW)	Among groups	−0.020	FCT = −0.000	.698
	Among populations within groups	0.090	FSC = 0.001	.121
	Within populations	99.930	FST = 0.001	.107
Scenario IV:Three groups (SSB, LYB, DSB, DYB) (ZSW, WTW, PTW, JLJW) (JSW, JZDW, ZSW, WTW, PTW, JLJW, YXW)	Among groups	1.310	FCT = 0.013	.277
	Among populations within groups	2.930	FSC = 0.030	.000
	Within populations	95.760	FST = 0.042	.000

### 3.2 Temporal variation in genetic diversity in wild populations of large yellow croaker

To understand whether the genetic diversity structure of large yellow croaker populations differs at different time points. The study conducted a comprehensive comparison and analysis of the genetic diversity fluctuations among wild populations of large yellow croaker over different years (Tables S1 and S2). Given the absence of genetic differentiation among various geographical populations, except for SSB and LYB, which was shown above to be more closely related to the cultured populations. Therefore, we conducted analyses on eight wild populations and two populations (SSB and LYB) affected by cultured individuals separately. As shown in Table S1, the results of this consolidation revealed that the haplotype diversity and nucleotide diversity of the large yellow croaker's wild populations across nine different years ranged from 0.962 (2017) to 0.994 (2016) for haplotype diversity, and from 0.406% (2019) to 0.465% (2017) for nucleotide diversity. As shown in Table S2, within the SSB and LYB populations, haplotype diversity ranged from 0.746 (2021) to 0.903 (2022), and nucleotide diversity ranged from 0.213% (2022) to 0.336% (2022). This observation strongly suggests that the level of genetic diversity among these wild populations remained notably stable and did not display significant fluctuations over the specified time frame. Between different years of geographic populations or among different geographic populations within the same year, the results indicate that there is no significant difference in their levels of genetic diversity (*p* = .360, *p* = .338) (Table S1). While to SSB, even though its nucleotide diversity was notably lower than that of the other wild populations, it too did not exhibit significant fluctuations across the five different years (Table S2).

### 3.3 The correlation among divergent aquatic regions and genetic diversity of large yellow croaker

In order to investigate the effects of divergent aquatic regions on the genetic structure of wild populations of large yellow croaker, this study selected 11 representative fishing locations (Figure 1), which can be categorized into three distinct marine environments: inner bay, inshore, and offshore. AMOVA from the two groups (Scenario I, the bay populations and other wild populations) identified only 3.340% of the variants to be present among groups, 2.200% of the variation among populations within groups and 94.460% of the variation within populations (Table 2). In the results of the three groups (Scenario III), only ‐0.020% of the variants were identified among groups, 0.090% of the variation among populations within groups, and 99.930% of the variation within populations (Table 2). These results indicate that differentiation within the wild populations of large yellow croaker is not influenced by habitat environments, except for the SSB and LYB populations. However, the analysis of genetic diversity within each population, as outlined in Table 3, revealed several noteworthy findings: First, significant distinctions were evident between the bay populations and the two open sea populations (*p* = .010). Second, there were notable variations in diversity levels among the four bay populations, accompanied by pronounced differentiation, underscoring distinct genetic structures within this group. Third, it is worth highlighting that there were no significant differentiation and genetic diversity level differences between the two bay populations, DSB and DYB, and the open sea populations, which suggests genetic similarities between these groups (Figure S2). Conversely, significant differences in differentiation levels and genetic diversity were observed between the other two bay populations (SSB and LYB) and the majority of populations in the open sea excluding the PTW and WTW populations in the adjacent sea area (Figure S2). Regarding the two open sea types, there were no significant differences in genetic diversity (*p* = .880) and genetic differentiation among the wild large yellow croaker populations, irrespective of the fishing gear used. This consistency held true not only within the same latitude range but also among the four inshore populations and seven offshore populations(*p* = .687) (Figure 1).

**TABLE 3:** Genetic diversity in different locations of the large yellow croakers.

	Area	Number of samples	Number of haplotypes, H	Haplotype diversity, Hd	Nucleotide diversity, θπ/%	Sum haplotype diversity, Hd	Sum nucleotide diversity, θπ/%
SSB	Bay	756	38	0.785	0.267	0.913	0.337
LYB	Bay	58	20	0.874	0.291		
DSB	Bay	84	59	0.985	0.393		
DYB	Bay	291	151	0.990	0.438		
ZSW	Inshore	53	23	0.962	0.444	0.985	0.442
WTW	Inshore	19	12	0.901	0.362		
PTW	Inshore	225	112	0.986	0.439		
JLJW	Inshore	111	78	0.988	0.458		
ZSW	Offshore	140	81	0.985	0.465	0.988	0.438
WTW	Offshore	255	127	0.983	0.411		
PTW	Offshore	10	9	0.978	0.396		
JLJW	Offshore	369	178	0.989	0.448		
JSW	Offshore	142	80	0.987	0.414		
JZDW	Offshore	89	70	0.993	0.448		
YXW	Offshore	121	82	0.989	0.459		

### 3.4 Genetic structure and relationships of large yellow croakers from different cultured populations

**FIGURE 3:**
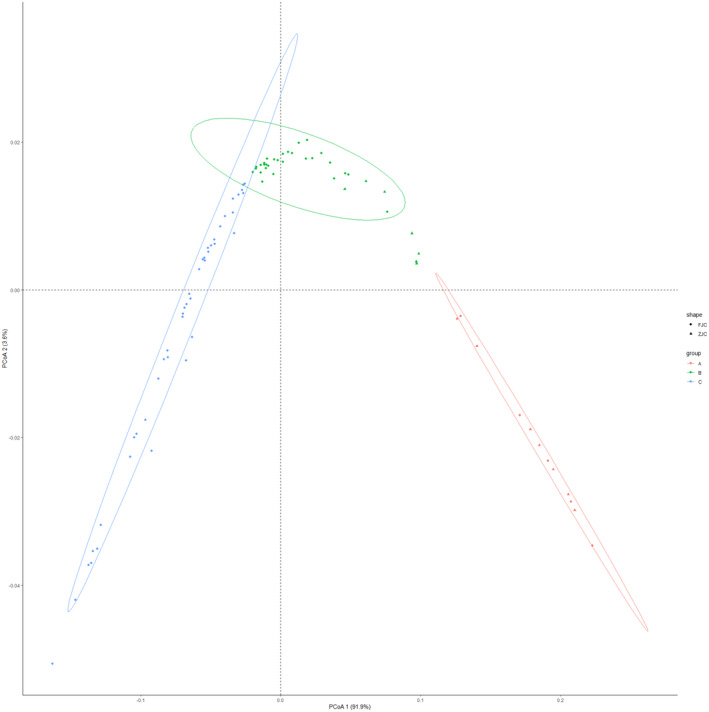
Principal coordinates analysis (PCoA) based on genetic distance showing three clusters within the cultured large yellow croaker. Use a diamond to indicate that the populations originated from the Fujian cultured population, use a triangle to indicate that the populations originated from the Zhejiang cultured population, and classify all the populations into three groups according to the different colors, where red was group A, green was group B, and blue was group C.

**FIGURE 4:**
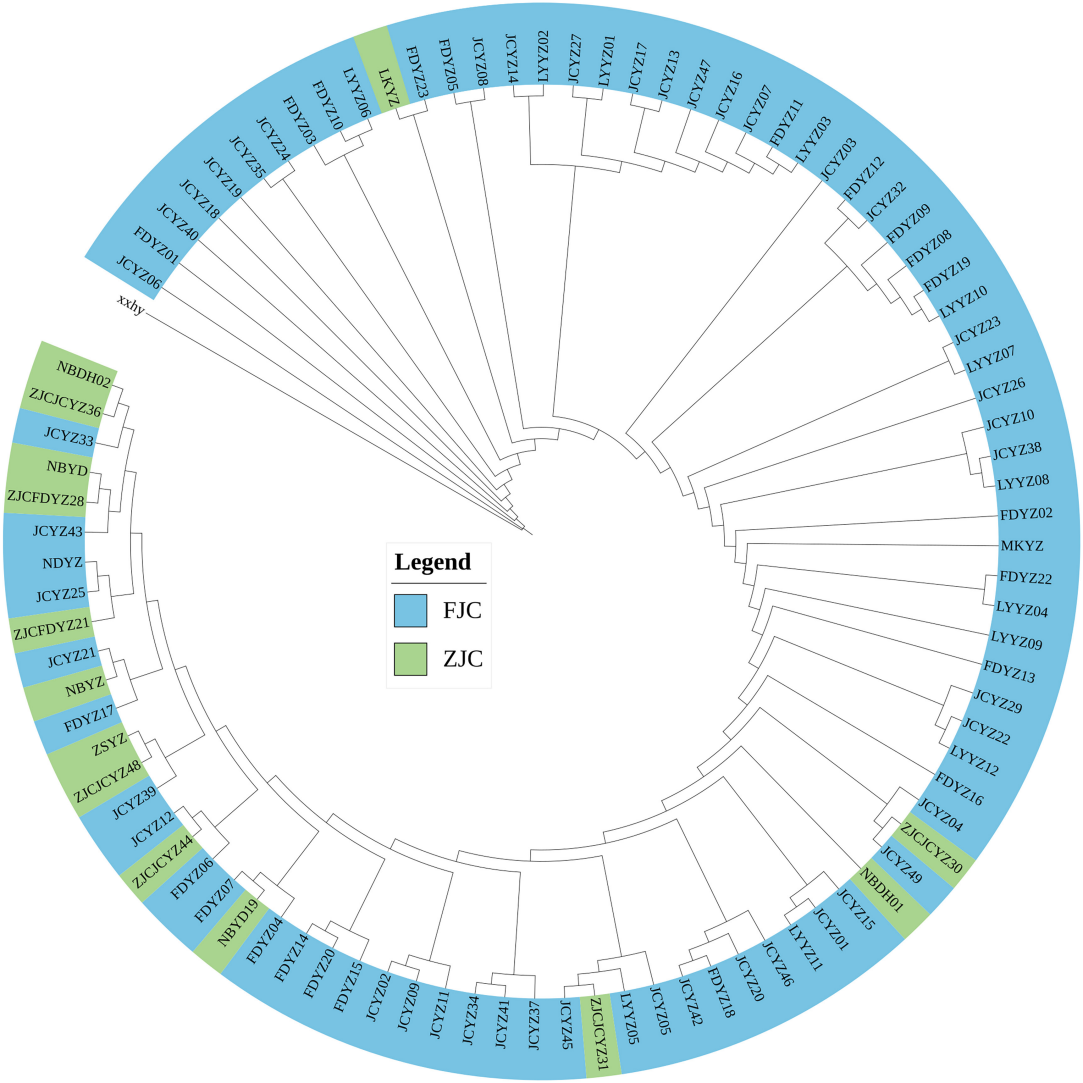
Neighbor‐joining phylogenetic tree based on genetic distance matrix representing the relationships between the different cultured populations. Blue color indicated that the cultured was from the Fujian cultured population, and green color indicated that the cultured was from the Zhejiang cultured population.

**FIGURE 5:**
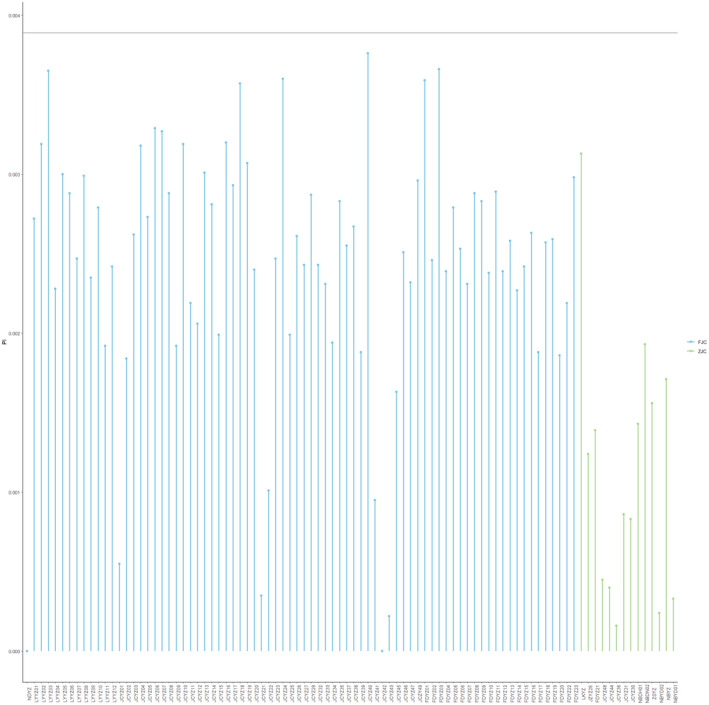
Nucleotide diversity of Fujian cultured and Zhejiang cultured populations. Blue color indicated that the cultured was from the Fujian cultured population, and green color indicated that the cultured was from the Zhejiang cultured population.

In order to provide valuable insights into the genetic relationships within and between different cultured populations of the cultured population of large yellow croaker, the principal coordinates analysis (PCoA) (Figure 3) and neighbor‐joining (NJ) phylogenetic tree (Figure 4) analyses were conducted. PCoA revealed that there was not significant genetic differentiation between the FJC and ZJC populations, despite them coming from different provinces. This suggests a shared ancestral founding population for the two regional populations, with some degree of differentiation within the whole cultured populations. The NJ tree also supported the lack of clear genetic structure between FJC and ZJC populations, aligning with the PCoA results. However, it did not exhibit the three main clusters seen in the PCoA analysis, indicating relatively weak differentiation within the cultured populations. Nucleotide diversity measures the level of genetic variation within a population. It is interesting to note that there were significant differences in nucleotide diversity between different cultured populations, regardless of whether they were from FJC or ZJC populations. For FJC populations, the nucleotide diversity ranged mainly between 0.002 and 0.003, samples from some populations in Fujian Province even approached the genetic diversity levels found in wild populations. In contrast, samples from ZJC populations exhibit a lower nucleotide diversity, primarily ranging from 0.000 to 0.002. This indicates that the genetic diversity of the ZJC is notably lower than that of the FJC (Figure 5).

## 4 DISCUSSION

Given the growing attention to global change, overfishing, and biodiversity loss, it is imperative to grasp the shifts and obstacles encountered by wildlife populations. In the conservation and management of marine fish, understanding genetic diversity, assessing populations' capacity to withstand and adapt to environmental shifts, and optimizing their evolutionary and genetic breeding potential are critical considerations (Eizaguirre & Baltazar‐Soares, 2014; Xu et al., 2015). Genetic diversity takes center stage in the conservation and management of animal genetic resources (Xu et al., 2015). Previous research indicates that overfishing contributes to the decline of genetic diversity in various marine fish species, resulting in a subsequent decrease in evolutionary potential and adaptive capacity. However, it is anticipated that the genetic drift accountable for this decline is likely to be weak in wide‐ranging and abundant species (Pinsky & Palumbi, 2014).

### 4.1 The genetic diversity of multiple geographic populations of large yellow croaker

The annual migration patterns of large yellow croaker play a crucial role in shaping its genetic structure (Zhang et al., 2023). During the reproductive season, the species migrates from the open sea to coastal areas, concentrating in inshore regions for spawning (Xu & Chen, 2011). This behavior facilitates gene exchange among spawning populations and contributes to the maintenance of genetic diversity within the species (Gaggiotti et al., 2009). The absence of significant differences among most wild populations, except for SSB and LYB, suggests the presence of a single panmictic genetic population of large yellow croaker. This pattern is consistent with observations in other marine fish species along the Chinese coast, where robust gene flow among populations has led to the emergence of panmictic populations (e.g., *Cirrhimuraena chinensis*, Li et al., 2014; *Lepturacanthus savala*, Gu et al., 2021; *Epinephelus awoara*, Yang et al., 2022). However, previous studies have reported some degree of differentiation among wild populations of large yellow croaker (Jiang et al., 2015; Kon et al., 2021). This discrepancy may be attributed to sampling errors, particularly in distinguishing between wild and cultured individuals which were released or escaped to nature environment. Our research found that the unique genetic profiles observed in various cultured populations underscore the importance of comprehensive sampling in accurately assessing genetic composition. Past studies have shown that inadequate sampling of cultured populations could lead to the misidentification of cultured populations as wild populations, thus leading to incorrect conclusions regarding population differentiation. Therefore, we believe careful consideration of sampling strategies is crucial for ensuring the accuracy of genetic assessments and population studies in large yellow croaker and other marine fish species.

### 4.2 Does the genetic diversity of the large yellow croaker change over time?

The large yellow croaker, once among China's top four marine species with a catch production of approximately 200,000 tons in the mid‐1970s (Liu & De Mitcheson, 2008), has witnessed a significant decline in fishery resources since 1975. This decline is attributed to the intensive exploitation of spawning and over‐wintering stocks (Liu & De Mitcheson, 2008). Previous studies have suggested that overfishing has contributed to a decrease in the genetic diversity of various marine fish species (Pinsky & Palumbi, 2014). Our study reveals that the haplotype diversity range of wild large yellow croaker in the early 20th century was between 0.979 and 0.993, with nucleotide diversity exceeding 0.004. The substantial haplotype diversity observed in the coastal regions of mainland China mirrors that of the majority of marine fishes, indicating sustained large effective population sizes over an extended period (e.g., *Terapon jarbua*, Liu et al., 2015; *Acanthogobius ommaturus*, Song et al., 2020; *Nemipterus bathybius*, Yi et al., 2021; *Trichiurus nanhaiensis*, Gu et al., 2022). This suggests that the genetic diversity of overfished species should be comparable to that of non‐depleted species, indicating minimal genetic impacts. Xu et al. (2022) integrated historical samples and illustrated that the genetic diversity of the wild large yellow croaker population has consistently maintained a high level over the past 60 years, as indicated by microsatellite markers (Ho = 0.740, He = 0.847) (Xu et al., 2022). These findings align closely with research conducted on other fish species, such as Atlantic salmon, Brown trout, and North Sea sole (Cuveliers et al., 2011; Hansen et al., 2002; Tessier & Bernatchez, 1999). In studies of iteroparous species capable of multiple reproductive events, genetic diversity typically does not decrease over time (Gajdárová et al., 2023; Jorde & Ryman, 2007). Analyses of these species also suggest that even when subjected to certain disturbances in their habitats, populations can maintain relatively high levels of genetic diversity (Wang et al., 2012; Xu et al., 2022). Therefore, the population can be considered to possess both highly abundant and stable genetic diversity, with the temporal stability of its genetic structure persisting for several decades. Our results indicate that the genetic diversity levels of the wild populations of the large yellow croaker remain significantly stable, showing no notable fluctuations within the specified time frame. This consistency with our previous research findings suggests that the genetic diversity of the large yellow croaker has not shown a significant decline due to overfishing and stocking releases (Yuan et al., 2021). In fish populations, natural (as opposed to anthropogenic) population turnover does not occur frequently unless the population resides in an extremely unstable environment (Hansen et al., 2002). We believe that the key factors maintaining genetic diversity in the large yellow croaker population are its relatively short generation time and larger population size (Gajdárová et al., 2023). Past research indicates that these potentially resilient species can recover to previous levels after experiencing overfishing pressure (Cuveliers et al., 2011; Hansen et al., 2002; Tessier & Bernatchez, 1999; Xu et al., 2022). Therefore, it is speculated that the current wild large yellow croaker population may still possess strong environmental adaptability, survival capability, and evolutionary potential (Lei et al., 2023).

### 4.3 Does the genetic variation of the large yellow croaker vary across different geographic locations?

Understanding the spatial distribution processes of fish populations is paramount for the conservation of fish species (Bartolino et al., 2017). The habitat distribution of the large yellow croaker encompasses three types: inner bay, inshore, and offshore. While previous studies, including our own, have revealed no significant genetic differentiation among wild populations from different geographical locations (Yuan et al., 2021), these studies did not consider the habitat divisions of the large yellow croaker during sampling. Therefore, we conducted sampling analysis across various habitats. Our findings indicate that the population of large yellow croaker did not show differentiation based on its habitat environment. However, populations belonging to the inner bay habitat, specifically those from SSB and LYB, showed differentiation from other wild populations. Interestingly, populations from DSB and DYB, also from an inner bay habitat, did not exhibit differentiation from other wild populations in different habitats. This suggests that the differentiation observed in the SSB and LYB populations from other populations is not solely attributed to the habitat. According to phylogenetic relationships, we observed a significantly high proportion of aquaculture‐derived haplotypes in the SSB (93.651%) and LYB (75.862%) populations. We believe this indicates differentiation between SSB and LYB populations and other wild populations. The SSB and LYB populations are located in Sansha Bay and Luoyuan Bay, respectively, in Fujian Province. These two regions are geographically adjacent, with Sansha Bay experiencing extensive large yellow croaker cage aquaculture and artificial stocking activities (Wang et al., 2012), while Luoyuan Bay lacks such activities (Li, 2018; Wu, 2020). We suggest that the decline in genetic diversity in the LYB population might stem from the introduction of aquaculture‐originated large yellow croaker migrating from Sansha Bay (Wang et al., 2012). The genetic diversity of DSB and DYB populations did not show significant differentiation from the wild populations (Figure S2). Since both DSB and DYB populations belong to inner bay populations, and the artificial stocking in their surrounding areas is relatively low or even absent, there are no individuals with genotypes related to the aquaculture population within these two populations. The variations in genetic diversity among different bay populations may be associated with stocking quantities and the behavior of large yellow croaker. The inner bay environment is considered more suitable for the aquaculture and survival of released individuals compared to the open sea environment (Santhanakumar et al., 2024). This result indicates that the survival rate of released large yellow croaker is not high in environments other than inner bay environments.

### 4.4 The impact of releases on the genetic diversity of wild populations and the genetic structure among populations within various cultured populations

Cultured release stands out as a widely utilized management strategy in fisheries, forestry, and wildlife management. However, the global concern lies in its adverse effects on wild populations (Kitada, 2018). Over the last 20 years, there has been a rapid increase in the practice of releasing aquaculture‐bred fish into the ocean to enhance marine fisheries. The Chinese government has implemented stock enhancement and release programs for more than two decades, aiming to restore the wild populations of the large yellow croaker in natural marine areas. These programs involve releasing a substantial quantity of artificially bred fish fry into the natural marine environment (Zhang et al., 2023). Stocking releases in aquaculture can lead to a decrease in the genetic diversity of wild populations, as previously reported in cultured marine fish such as *Salmo salar L*. (Machado‐Schiaffino et al., 2007) and *Acanthopagrus schlegelii* (Shan et al., 2020). Based on our findings and previous research, in addition to the SSB and LYB populations, there is significant genetic differentiation between cultured and wild populations (Yuan et al., 2021). In the initial stages of large yellow croaker aquaculture in China, large‐scale factory nursery practices were employed to protect the fisheries resources of large yellow croaker. With an increase in the number of cultured populations, some facilities began targeted breeding programs for large yellow croaker (Xu et al., 2022). These programs encompassed genetic improvement and selection (Xu et al., 2015), selection for high growth rates (Yan et al., 2022; Zhou et al., 2019), and selection for disease resistance (Bai et al., 2020; Kong et al., 2019). These processes significantly altered the genetic diversity of cultured large yellow croaker (Figures 3 and 4). We suggest this is a result of cultured release individuals' inability to adapt to survive in the wild environment (Figure 5). The previous study revealed that domestication leads to the relaxation of purifying selection, resulting in increased genetic loads. Consequently, cultured fish, such as the large yellow croaker, may face a selective disadvantage when cultured juveniles are released into the wild (Yuan et al., 2021). Domestication is the process through which fish adapt to the human environment and its limitations (Milla et al., 2021). In the past 35 years, various cultured populations have been subjected to directional selection for a widening range of economically significant traits, resulting in genetic differentiation among different aquaculture populations (Kon et al., 2021). The main distributions for cultured large yellow croaker are primarily located in Zhejiang and Fujian provinces. The results of this study suggest that, similar to many cultured fish species, their genetic diversity is lower compared to wild populations (An et al., 2011; Yang et al., 2019). However, intriguingly, there is evidence of differentiation within the cultured populations in Zhejiang and Fujian provinces, as depicted in Figure 5. We suggest that this discrepancy in direction of domestication among different cultured populations has led to these results. Additionally, the Fujian cultured population exhibits higher genetic diversity and genotype richness compared to the Zhejiang population. We suggest that some individuals from the Zhejiang population may have originated from the cultured population in Fujian rather than from wild populations.

## AUTHOR CONTRIBUTIONS

**Ji‐Xiang Yao:** Data curation (lead); formal analysis (lead); methodology (lead); software (lead); writing – original draft (lead). **Hung‐Du Lin:** Formal analysis (equal); methodology (equal); supervision (equal); writing – original draft (lead); writing – review and editing (equal). **Li‐Sheng Wu:** Conceptualization (equal); formal analysis (equal); methodology (equal); software (equal); writing – review and editing (equal). **Li‐Na Wu:** Investigation (equal); methodology (equal); software (equal). **Ji‐Gui Yuan:** Data curation (equal); methodology (equal); software (equal). **Shao‐Xiong Ding:** Conceptualization (lead); funding acquisition (lead); investigation (lead); project administration (lead); supervision (lead); writing – review and editing (lead).

## CONFLICT OF INTEREST STATEMENT

The authors declare no conflict of interest.

## Supporting information


Figure S1.

Figure S2.

Table S1.

Table S2.

Table S3.

## Data Availability

The data that support the findings of this study are available in Genbank (https://www.ncbi.nlm.nih.gov/), reference number (cyt *b*: MW233899–MW233949 and RAG‐1: MW233950–MW234070).
