# Supplementary figures and images for: Stability of population genetic structure in large yellow croaker (Larimichthys crocea): Insights from temporal, geographical factors, and artificial restocking processes
Source: Ecol Evol. 2024 Aug 27;14(8):e70207. doi: 10.1002/ece3.70207 (PMC11347937; doi:10.1002/ece3.70207)

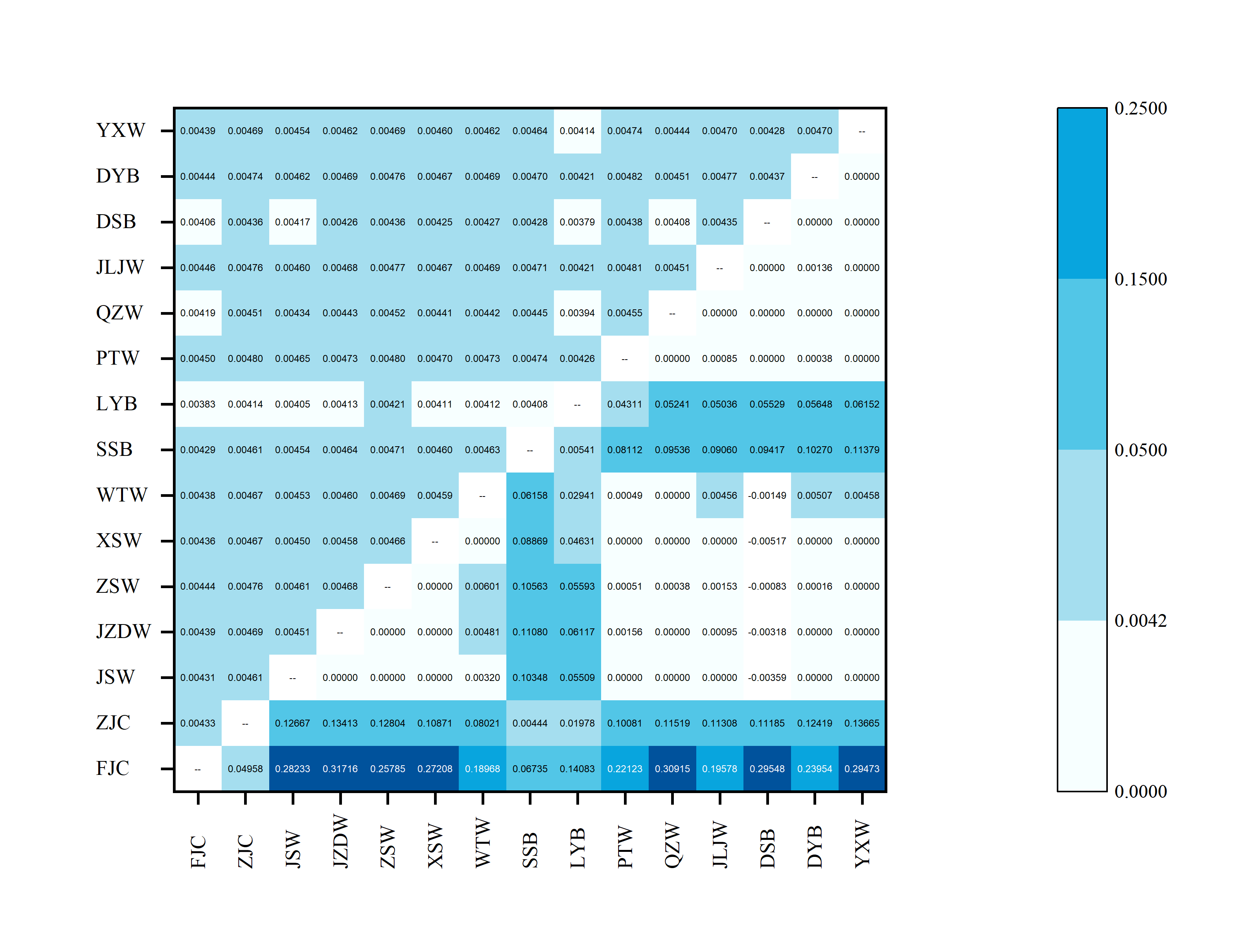

Supplement: Supplementary file 1 — Figure S1. [file ECE3-14-e70207-s003.png]

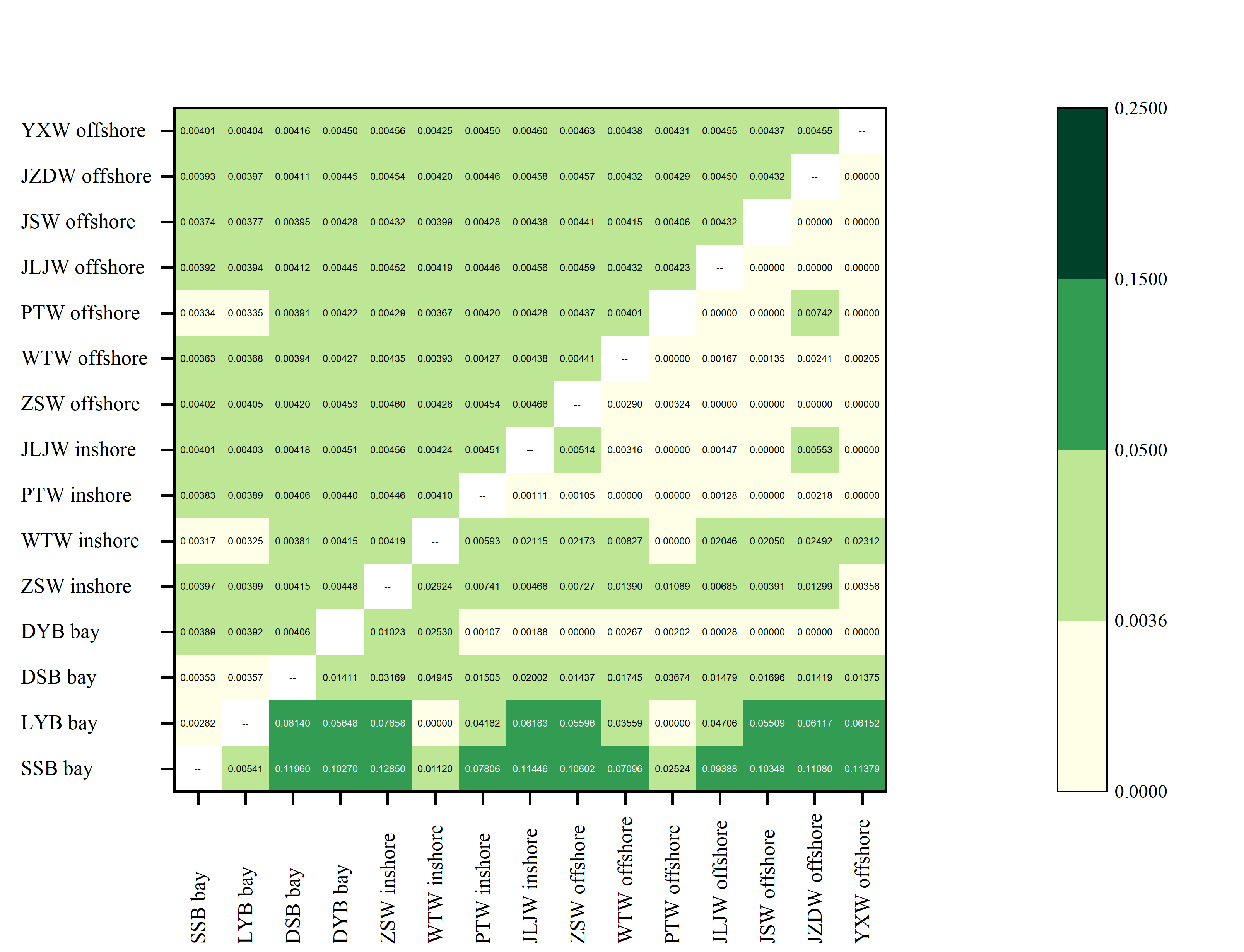

Supplement: Supplementary file 2 — Figure S2. [file ECE3-14-e70207-s001.png]
