# Supplementary material for: Stability of population genetic structure in large yellow croaker (Larimichthys crocea): Insights from temporal, geographical factors, and artificial restocking processes
Source: Ecol Evol. 2024 Aug 27;14(8):e70207. doi: 10.1002/ece3.70207 (PMC11347937; doi:10.1002/ece3.70207)
Supplement: Supplementary file 3 — Table S1. [file ECE3-14-e70207-s005.docx]

**Supplementary figure captions**

**Figure S1.** Genetic distance (above diagonal) and Pairwise *F*_ST_ (below diagonal) among 2 cultured population and 13 wild populations of the large yellow croaker.

**Figure S2.** Genetic distance (above diagonal) and pairwise *F*_ST_ (below diagonal) among different locations of the large yellow croaker.

**Table S1** Nucleotide diversity of different wild populations that ranked according to years of the large yellow croaker.

| Year | Nucleotide diversity of populations, θπ/% | | | | | | | | Number of Haplotypes, H | Haplotype diversity, Hd | Nucleotide diversity, θπ/% |
| --- | --- | --- | --- | --- | --- | --- | --- | --- | --- | --- | --- |
|  | JSW | ZSW | WTW | PTW | QZW | JLJW | DYB | YXW |  |  |  |
| 2008 | 0.460 | NA | NA | NA | NA | NA | NA | NA | 17 | 0.981 | 0.460 |
| 2016 | NA | NA | NA | NA | NA | NA | NA | 0.441 | 24 | 0.994 | 0.441 |
| 2017 | NA | NA | NA | NA | NA | 0.462 | NA | NA | 24 | 0.962 | 0.462 |
| 2018 | NA | 0.460 | 0.443 | NA | NA | 0.433 | NA | 0.476 | 119 | 0.990 | 0.455 |
| 2019 | 0.384 | 0.438 | 0.405 | NA | NA | NA | NA | NA | 85 | 0.983 | 0.406 |
| 2020 | NA | NA | 0.403 | 0.505 | NA | 0.423 | NA | NA | 99 | 0.987 | 0.459 |
| 2021 | 0.402 | NA | 0.390 | 0.407 | 0.425 | 0.465 | 0.424 | NA | 177 | 0.984 | 0.410 |
| 2022 | 0.446 | 0.473 | 0.433 | 0.365 | 0.438 | 0.425 | 0.440 | 0.446 | 404 | 0.992 | 0.435 |
| 2023 | NA | NA | 0.417 | 0.392 | 0.367 | 0.426 | 0.440 | NA | 86 | 0.984 | 0.407 |
| Total | 0.414 | 0.460 | 0.410 | 0.436 | 0.421 | 0.436 | 0.438 | 0.459 |  |  |  |
