# Supplementary material for: Stability of population genetic structure in large yellow croaker (Larimichthys crocea): Insights from temporal, geographical factors, and artificial restocking processes
Source: Ecol Evol. 2024 Aug 27;14(8):e70207. doi: 10.1002/ece3.70207 (PMC11347937; doi:10.1002/ece3.70207)
Supplement: Supplementary file 4 — Table S2. [file ECE3-14-e70207-s002.docx]

**Table S2** Nucleotide diversity of cultured-influenced different wild populations that ranked according to years of the large yellow croaker.

| Year | Nucleotide diversity of populations, θπ/% | | Number of Haplotypes, H | Haplotype diversity, Hd | Nucleotide diversity, θπ/% |
| --- | --- | --- | --- | --- | --- |
|  | SSB | LYB |  |  |  |
| 2017 | 0.294 | NA | 10 | 0.865 | 0.294 |
| 2019 | 0.296 | NA | 27 | 0.829 | 0.296 |
| 2020 | 0.247 | NA | 9 | 0.792 | 0.247 |
| 2021 | 0.260 | NA | 19 | 0.746 | 0.260 |
| 2022 | 0.213 | 0.336 | 17 | 0.903 | 0.310 |
| 2023 | NA | 0.266 | 12 | 0.861 | 0.266 |
| Total | 0.268 | 0.291 | 48 | 0.792 | 0.269 |
