# Supplementary material for: Stability of population genetic structure in large yellow croaker (Larimichthys crocea): Insights from temporal, geographical factors, and artificial restocking processes
Source: Ecol Evol. 2024 Aug 27;14(8):e70207. doi: 10.1002/ece3.70207 (PMC11347937; doi:10.1002/ece3.70207)
Supplement: Supplementary file 5 — Table S3. [file ECE3-14-e70207-s004.docx]

**Table S3** Detailed table of large yellow croaker sample collection.

| Location | Code name | Sample size | Year of sampling | Sampling location | Longitude and latitude |
| --- | --- | --- | --- | --- | --- |
| Zhejiang Cultured | ZJC | 415 | 2017-2019,2021 | Xiangshan Port, Ningbo, Zhejiang Province and Zhoushan City, Zhejiang Province. Fuding City, Fujian Province and Jiaocheng District, Ningde City, Fujian Province. | \ |
| Fujian Cultured | FJC | 2372 | 2017-2019 | Fuding City, Fujian Province, Jiaocheng District, Ningde City, Fujian Province, Luoyuan County, Fuzhou City, Fujian Province | \ |
| Jiangsu | JSW | 142 | 2006, 2008, 2019, 2021, 2022 | Lüsi Fishing Ground and south of yellow sea | 32°00ˊ～34°00ˊN, West of 125°E |
| Jeju Island | JZDW | 89 | 2022 | Near the China-Korea border | 27°00ˊ～30°40ˊN, 121°57.4ˊ~128°32.2ˊE |
| Zhoushan Island | ZSW | 193 | 2018, 2019, 2022 | China Zhoushan fishing ground | 30°44.07ˊ～32°00ˊN, 122°～128°00ˊE |
| Yushan Islands | XSW | 99 | 2019-2022 | Near Yushan Islands, Ningbo, Zhejiang Province | \ |
| Wenzhou and Fuding | WTW | 306 | 2020-2023 | 26°00ˊ～29°30ˊN, West of 125°00ˊE | 26°00ˊ～29°30ˊN, West of 125°00ˊE |
| Sansha Bay | SSB | 756 | 2017, 2019-2023 | Sansha Bay, Fujian Province | \ |
| Luoyuan Bay | LYB | 58 | 2022-2023 | Luoyuan Bay, Fujian Province | \ |
| Haitan Island | PTW | 243 | 2020-2023 | Sea area around Haidan Island | 24°30ˊ～28°16ˊN, West of 121°30ˊE |
| Quanzhou | QZW | 67 | 2021-2023 | Near Quanzhou, Fujian Province | 24°30ˊ～24°54ˊN, 118°24ˊ～118°43ˊE |
| Kinmen Island | JLJW | 667 | 2017, 2018, 2020-2023 | Southern Fujian | 24°30ˊ～24°54ˊN, 118°24ˊ～118°43ˊE |
| Dongshan Bay | DSB | 84 | 2020-2022 | Dongshan Bay, Fujian Province | \ |
| Daya Bay | DYB | 291 | 2021-2023 | Daya Bay, Huizhou, Guangdong Province | \ |
| Leizhou Peninsula | YXW | 121 | 2016, 2018, 2022 | Near Leizhou Peninsula, Zhanjiang, Guangdong Province | 18°30ˊ～21°06ˊN, 110°30ˊ～110°55ˊˊE |
